# Sexual Dysfunction in the Life Cycle of Women: Implications for Psychological Health

**DOI:** 10.3390/healthcare13111268

**Published:** 2025-05-27

**Authors:** Samet Kırat

**Affiliations:** Department of Gynecology and Obstetrics, Faculty of Medicine, Kafkas University, Kars 36000, Turkey; sametkirat1989@hotmail.com

**Keywords:** anxiety, depression, emotional stress, menopause, sexual dysfunction

## Abstract

**Objective:** Sexual dysfunction (SD) is a prevalent but frequently overlooked condition that adversely affects women’s quality of life and psychological well-being. This study aimed to examine the relationship between SD and depression, anxiety, and stress levels during premenopausal, pregnancy, and postmenopausal. **Methods:** This prospective, cross-sectional study included 300 women aged 18–70 who presented with SD symptoms to a tertiary care gynecology outpatient clinic. Participants were categorized into premenopausal, pregnant, and postmenopausal groups. Sexual function was assessed using the Female Sexual Function Index (FSFI), and psychological status was evaluated with the Depression Anxiety and Stress Scale (DASS-21). Univariate and multivariate logistic regression analyses were conducted to identify risk factors associated with SD. **Results:** The results showed that SD prevalence varied across life stages, with the highest rate (96%) observed in postmenopausal women. Significant decreases were found in all FSFI subscales, particularly lubrication and orgasm, during the postmenopausal period (*p* < 0.001). Although DASS-21 total scores did not differ significantly between groups (*p* = 0.227), severe stress was more prevalent in premenopausal women (*p* = 0.018). Univariate logistic regression revealed that older age, higher parity, and menopause increased SD risk (*p* < 0.001), while employment (*p* = 0.006), higher education (*p* = 0.012), and pregnancy (*p* < 0.001) were protective factors. Multivariate analysis identified parity as the only independent variable significantly increasing SD risk (*p* = 0.011). Weak but significant negative correlations were found between FSFI total score and DASS-21 total (r = −0.137, *p* = 0.018), anxiety (r = −0.135, *p* = 0.019), and depression (r = −0.176, *p* = 0.002) scores. **Conclusion:** These findings highlight the importance of individualized assessment and treatment approaches for women’s sexual health across different life stages, considering the influence of various biological, psychological, and social factors.

## 1. Introduction

Sexual health is an essential component of overall quality of life and well-being; however, it remains one of the most taboo and least discussed areas of health, especially for women. Throughout the life cycle from puberty to menopause, women’s sexual functioning is influenced by many biological, psychological, and social factors. Changes in hormone levels, life events such as childbirth or relationship transitions, and societal attitudes towards female sexuality are among the main factors that shape sexual experiences [1]. Despite these variables, sexual dysfunction (SD) in women is still underreported and often untreated [2].

SD in women encompasses multifaceted problems, such as decreased sexual desire, arousal problems, orgasm difficulties, and painful intercourse [3]. These problems can occur at any time of life and are often intertwined with mental health problems such as anxiety, depression, and low self-esteem. Hormonal changes, especially during menstruation, pregnancy, postpartum, and menopause, significantly affect sexual function [4]. When psychological stressors and relationship dynamics are added to these biological changes, a complex picture emerges that affects both women’s sexuality and emotional health.

A bidirectional relationship between SD and psychological health has been shown [1]. On one hand, mental problems such as depression and anxiety reduce sexual desire and satisfaction; on the other hand, persistent sexual problems can lead to feelings of inadequacy, relational conflicts, and deterioration in mental health [3]. Silence and shame surrounding female sexuality further deepen this cycle by inhibiting help-seeking behaviors [2]. Recent studies suggest that postmenopausal women are at a more pronounced risk in this respect due to age-related physiological changes, comorbid disease burden, and increased psychosocial vulnerabilities [5,6]. This necessitates the adoption of age-specific and holistic intervention approaches in the assessment of SD.

The aim of this study was to comparatively evaluate the relationship between SD and depression, anxiety, and stress levels in premenopausal, pregnant, and postmenopausal women. The study aims to reveal the effects of physiological and psychosocial changes that women face at different life stages on sexual function and mental state from a holistic perspective. In this context, in line with the objectives of the study, it was hypothesized that depression, anxiety, and stress levels would differ significantly among premenopausal, pregnant, and postmenopausal women and that SD would be more strongly associated with psychological distress due to increased hormonal and psychosocial changes, especially during pregnancy and the postmenopausal period.

## 2. Materials and Methods

### 2.1. Study Design

This prospective cross-sectional study included 300 women who met the inclusion criteria and presented to the Department of Obstetrics and Gynecology at the Kafkas University Faculty of Medicine between April and June 2024.

### 2.2. Inclusion Criteria

The study included women between the ages of 18–70, married, sexually active, and willing to participate in the study, who showed signs and symptoms of SD (such as decreased sexual desire, arousal difficulties, vaginal dryness, inability to orgasm, and pain during sexual intercourse).

### 2.3. Exclusion Criteria

Patients with active psychiatric illness, comorbidities, medications that may affect sexual function (for example, SSRIs, antipsychotics, estrogen and/or androgen therapy, beta-blockers, etc.), recent vaginal infections, and history of pelvic surgery were excluded from the study. These exclusion criteria were applied to eliminate potential confounding variables that could directly affect sexual function and psychological status. With this approach, we aimed to obtain a more homogeneous sample, free from the effects of gynecological, psychiatric, or surgical history, and to analyze the differences between reproductive periods in a more reliable and comparable way.

### 2.4. Covariates and Descriptive Data

Demographic and health-related characteristics analyzed were age, number of gravida, number of parities, number of abortions, smoking, employment status, education level (primary, secondary, or higher education), and yearly income level (low, medium, high). These variables were accepted as covariates in intergroup comparisons and multivariate statistical analyses.

### 2.5. Group Selection

Participants were divided into three groups according to their reproductive periods: premenopausal, pregnancy, and postmenopausal. Within each group, subgroups were formed by those with and without SD. Comparisons were made both between groups according to the reproductive period and between subgroups according to the presence of SD.

### 2.6. Questionnaire Scales

#### 2.6.1. The Female Sexual Function Index (FSFI)

The FSFI is a 19-item Likert-type scale with proven validity and reliability developed to assess women’s sexual function in the last month. It includes six subscales: sexual desire (e.g., Over the past 4 weeks, how often did you feel sexual desire or interest?), sexual arousal (e.g., Over the past 4 weeks, how often did you feel sexually aroused during sexual activity or intercourse?), lubrication (e.g., Over the past 4 weeks, how often did you become lubricated (‘wet’) during sexual activity?), orgasm (e.g., Over the past 4 weeks, how often did you reach orgasm during sexual intercourse or activity?), sexual satisfaction (e.g., Over the past 4 weeks, how satisfied have you been with your sexual life?), and pain (e.g., Over the past 4 weeks, how often did you experience pain or discomfort during vaginal penetration?). Responses were graded from 1 (never) to 5 (always), and participants who had not had sexual intercourse in the previous month answered the relevant items on a scale of 0. The total score ranges between 2 and 36, and scores below 26.55 are considered to indicate the presence of SD [7].

#### 2.6.2. Depression Anxiety and Stress Scale (DASS)-21

The DASS-21 is a self-report-based, validated, and reliable brief psychological assessment tool used to assess depression, anxiety, and stress levels of individuals. The scale consists of three subscales of seven items each: depression (e.g., I felt like I could not feel any positive emotions), anxiety (e.g., I felt like panicking), and stress (e.g., I had difficulty relaxing). Each item has a four-point Likert-type response format, ranging from 0 “not at all suitable for me” to 3 “very suitable for me” to assess the individual’s feelings and thoughts in the last week. The scores obtained for each sub-dimension range from 0 to 21, and these scores are categorized according to certain ranges to rate the mental state of the individual. Accordingly, 0–4 points for depression is considered normal, 5–6 mild, 7–10 moderate, 11–13 severe, and 14 and above very severe; 0–3 points for anxiety is considered normal, 4–5 mild, 6–7 moderate, 8–9 severe, and 10 and above very severe; and 0–7 points for stress is considered normal, 8–9 mild, 10–12 moderate, 13–16 severe, and 17 and above very severe [8].

### 2.7. Statistical Analysis

Data analysis was performed using SPSS for Windows (version 24.0; SPSS Inc., Chicago, IL, USA). Continuous variables were summarized as median (minimum-maximum), and categorical data were summarized as frequency and percentage. The assumption of normal distribution was evaluated with the Kolmogorov–Smirnov test. The Mann–Whitney U and Kruskal–Wallis tests were used for comparisons between groups that were not normally distributed. Categorical variables were analyzed with the chi-square (χ^2^) test.

To determine the predictive variables affecting the presence of SD, variables found to be significant (*p* < 0.05) in univariate analyses were included in multivariate logistic regression analysis. The relationship between DASS-21 and FSFI scores was evaluated by Pearson correlation analysis. Odds ratios (OR), 95% confidence intervals (CI), and *p*-values were reported in the regression analysis. The significance level was accepted as *p* < 0.05 in all statistical analyses.

### 2.8. Ethics Approval and Consent to Participate

This study was approved by the Non-Interventional Clinical Research Ethics Committee of the Kafkas University Faculty of Medicine (27 March 2024, 80576354-050-99/426). Data collection was conducted in accordance with the Declaration of Helsinki. Participants were informed in detail about the purpose of the study, the forms used, and the principles of confidentiality; it was explained that the data would be collected anonymously and would be used only for scientific purposes. Individuals who met the eligibility criteria were informed by the research assistant in the outpatient clinic waiting area and invited to participate in the study. Those who agreed to participate completed the forms in a quiet and private room after written informed consent was obtained. The average form-filling time was 15 min, and participants with literacy difficulties or physical disabilities were supported by a trained research assistant. In total, 360 women were contacted; 300 participated in the study and completed the forms completely (response rate: 83.3%). The 60 women who did not meet the eligibility criteria or refused to participate were excluded. Since the forms were completed in a single session, there was no loss to follow-up.

## 3. Results

### 3.1. Demographic Characteristics, FSFI and DASS-21 Results of the Total Cohort

The age of the 300 women ranged between 18 and 70, with a median age of 36. According to the findings obtained from the FSFI scale, SD was detected in 240 women (80%). The median total score of the DASS-21 scale was 9 (0–48). The median anxiety score was 3 (0–16), the median depression score was 2 (0–18), and the median stress score was 3 (0–18) (Table 1).

### 3.2. Comparison of Those With and Without Sexual Dysfunction

The median age was significantly higher in patients with SD than in those without SD (*p* < 0.001). Gravida (*p* < 0.001) and parity (*p* < 0.001) were significantly higher in women with SD compared to women without SD. The number of employed women was significantly higher in women without SD than in those with SD (*p* = 0.009). The number of primary school graduates was significantly higher in women with SD than in those without SD (*p* = 0.024). Total scores obtained from the DASS-21 scale (*p* = 0.194), anxiety scores (*p* = 0.170), and stress scores (*p* = 0.542) did not show a statistically significant difference between the groups with and without SD. However, depression scores were significantly higher in the group with SD than in the group without SD (*p* = 0.013) (Table 2).

### 3.3. Comparison of Demographic and Obstetric Characteristics According to Premenopausal, Pregnancy and Postmenopausal Periods

The number of gravida (*p* < 0.001) and parity (*p* < 0.001) were significantly higher in the postmenopausal period. The number of working women was significantly higher during pregnancy (*p* = 0.008). Education level was significantly higher during pregnancy (*p* < 0.001). Socioeconomic status was significantly higher in the postmenopausal period (*p* = 0.012). Smoking was significantly higher in the premenopausal period (*p* = 0.045) (Table 3).

### 3.4. Comparison of FSFI Scale Results According to Premenopausal, Pregnancy and Postmenopausal Periods

Among women with SD, 32.1% were premenopausal, 27.9% were pregnant, and 40% were postmenopausal (*p* < 0.001). In the postmenopausal period, the scores for all subscales of the FSFI were significantly lower (*p* < 0.001) (Table 4). The significantly lower FSFI scores in the postmenopausal period and the SD rate reaching 96% support our first hypothesis that sexual function varies across different stages of life.

### 3.5. Comparison of DASS-21 Scale Results According to Premenopausal, Pregnancy and Postmenopausal Periods

**Figure 1:**
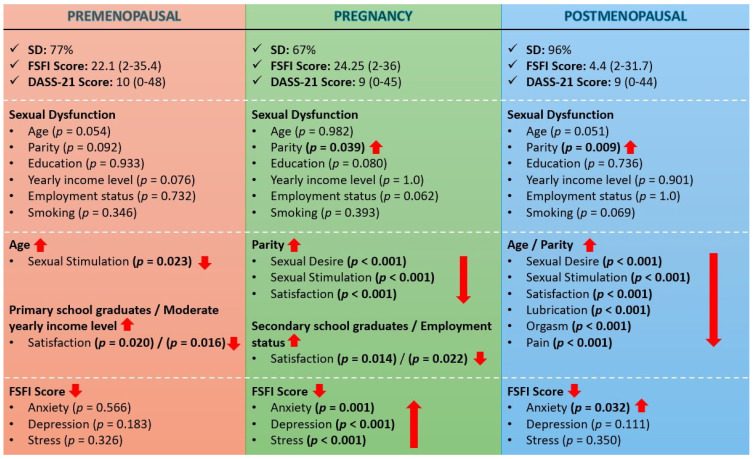
A comparative summary of findings in premenopausal, pregnancy, and postmenopausal periods is presented. Significant associations specific to each period are highlighted with red arrows and *p*-values.

The total scores obtained from the DASS-21 scale (*p* = 0.227), anxiety scores (*p* = 0.773), depression scores (*p* = 0.144), and stress scores (*p* = 0.080) were similar in all three groups. However, the rate of severe stress was significantly higher in the premenopausal period (*p* = 0.018) (Table 5). A comparative summary of findings in premenopausal, pregnancy, and postmenopausal periods is presented in Figure 1.

### 3.6. Univariate and Multivariate Analysis for Risk Factors for SD

According to univariate logistic regression analysis, factors that significantly increased the risk of developing SD included age (OR: 1.075; 95% CI: 1.045–1.106; *p* < 0.001), parity (OR: 1.689; 95% CI: 1.382–2.065; *p* < 0.001), and menopause (OR: 9.333; 95% CI: 3.277–26.584; *p* < 0.001). In contrast, being employed (OR: 0.347; 95% CI: 0.162–0.741; *p* = 0.006), educational level (OR: 0.579; 95% CI: 0.377–0.888; *p* = 0.012), and being pregnant (OR: 0.317; 95% CI: 0.177–0.567; *p* < 0.001) were found to significantly reduce the risk of developing SD (Table 6).

According to the multivariate logistic regression analysis, parity was the only factor that significantly increased the risk of developing SD (OR: 1.421; 95% CI: 1.085–1.861; *p* = 0.011) (Table 7).

### 3.7. Evaluation of the Relationship Between FSFI and DASS-21 Scale Scores

According to the Spearman correlation analysis results, a weak but statistically significant negative correlation was found between the DASS-21 total score and the FSFI total score (r = −0.137, *p* = 0.018). When analyzed by subscales, it was determined that there were weak but statistically significant negative relationships between anxiety (r = −0.135, *p* = 0.019), depression (r = −0.176, *p* = 0.002), and FSFI scores. On the other hand, although a negative relationship was observed between stress subscale and FSFI score, this relationship was not statistically significant (r = −0.098, *p* = 0.090). These findings suggest that psychological well-being may affect female sexual functioning.

## 4. Discussion

In this study, SD ratios were determined based on the FSFI scores of 300 women in the premenopausal, pregnancy, and postmenopausal periods, and the relationship between these scores and DASS-21 was examined. The SD rate was found to be significantly higher in the postmenopausal period (*p* < 0.001), and a significant decrease was found in all sexual function areas, especially in the lubrication and orgasm subscales (*p* < 0.001). Although the DASS-21 total scores did not differ significantly between the groups (*p* = 0.227), the proportion of women experiencing severe stress was higher in the premenopausal group (*p* = 0.018). In univariate logistic regression analysis, older age, higher parity, and menopause were found to be the main factors increasing the risk of SD (*p* < 0.001), whereas working life (*p* = 0.006), higher education level (*p* = 0.012), and pregnancy (*p* < 0.001) were found to be protective factors. In the multivariate analysis, only parity stood out as an independent variable that significantly increased the risk of SD (*p* = 0.011).

According to studies conducted in the premenopausal period, the prevalence of SD varies between 22.7% and 72.2% [9]. Decreased sexual desire and orgasmic disorders have been reported to be more common in women who have had three or more births [10]. Smoking has been shown to negatively affects sexual function, particularly in areas such as sexual desire, lubrication, orgasm, and satisfaction [11]. It has been reported that women with higher levels of education may experience a decrease in areas such as desire, satisfaction, and orgasm due to relational problems, while economic difficulties and relational stress increase the risk of SD in women with low socioeconomic status [12]. In our study, the SD rate in the premenopausal period was 77%. There was no statistically significant difference between the groups with and without SD in terms of age (*p* = 0.054), parity (*p* = 0.092), educational level (*p* = 0.933), socioeconomic level (*p* = 0.076), and smoking (*p* = 0.346). However, a significant decrease in sexual arousal scores was observed with increasing age (*p* = 0.023). In addition, it was found that sexual satisfaction scores were higher in individuals who had graduated from primary school (*p* = 0.020) and those with moderate socioeconomic status (*p* = 0.016).

Studies examining the effect of FSFI subscales on DASS-21 scores in the premenopausal period found that desire, arousal, lubrication, orgasm, and satisfaction subscales were negatively correlated with depression, anxiety, and stress subscales of DASS-21 [13]. In a study conducted during the COVID-19 pandemic, it was reported that all subscales of the FSFI were negatively correlated with DASS-21 scores, and anxiety played a significant role in this decrease [14]. Similarly, the desire, lubrication, and orgasm subscales have been shown to be negatively associated with somatization and depression scores [15], and the desire, arousal, and satisfaction subscales with depression and anxiety [16]. It was reported that improvement in the satisfaction and desire subscales after counseling with the PLISSIT model reduced the depression and stress scores of the DASS-21 [17]. In our study, no significant relationship was found between FSFI scores and DASS-21 subscales of anxiety (*p* = 0.566), depression (*p* = 0.183), and stress (*p* = 0.326) scores in premenopausal women. The fact that the individuals in our study were selected from a community-based sample who did not apply for psychosexual problems and that their DASS-21 scores were below the clinical thresholds may be one of the main reasons for the lack of a statistically significant relationship. In addition, factors such as social taboos about sexual health, low level of awareness, and response bias in self-report scales may have made it difficult to reveal the relationship between psychological status and sexual function.

The prevalence of SD in pregnancy varies between 57% and 92% [18,19,20,21]. It has been reported that FSFI scores are higher in women with higher levels of education, whereas a decrease in desire, arousal, orgasm, and satisfaction is observed in women with low socioeconomic status and working women, and this increases the prevalence of SD [22,23]. It has been reported that smoking during pregnancy negatively affects the desire and satisfaction subscales [22], and a history of miscarriage decreases FSFI scores in relation to depression [24]. It has been reported that there was a significant decrease in all subscales in the third trimester [19,21]; in particular, the satisfaction, lubrication, and pain subscales decreased to the lowest levels [19,24,25]. In our study, the SD rate during pregnancy was 67%. There were no significant differences between the groups with and without SD in terms of age (*p* = 0.982), educational level (*p* = 0.080), employment status (*p* = 0.062), socioeconomic status (*p* = 1.0), and smoking (*p* = 0.393). However, parity was significantly higher in the group with SD (*p* = 0.039), and a significant decrease was observed in sexual desire (*p* < 0.001), arousal (*p* < 0.001), and satisfaction (*p* = 0.007) scores as parity increased. In addition, sexual satisfaction scores were lower among secondary school graduates (*p* = 0.014) and working women (*p* = 0.022).

Studies conducted during pregnancy have shown that FSFI subscales are negatively correlated with DASS-21 scores. In particular, decreases in satisfaction and lubrication have been reported to increase depression [24,26]. It has been reported that SD increases stress scores as pregnancy progresses, and SD and anxiety become more pronounced in the third trimester [25]. It was observed that the subscales of sexual desire and sexual satisfaction showed a strong negative relationship with depression and stress scores, and this relationship deepened as pregnancy progressed [26]. It was reported that somatization and anxiety increased with decreased sexual function, while counseling interventions improved satisfaction and desire scores, and decreased depression and stress [27]. In our study, statistically significant and negative correlations were found between FSFI scores and anxiety (*p* = 0.001), depression (*p* < 0.001), and stress (*p* < 0.001) subscale scores of the DASS-21 scale in pregnant women. The negative correlations observed between FSFI scores and DASS-21 subscales during pregnancy indicate a stronger association between psychological distress and sexual dysfunction in this period, supporting our second hypothesis that psychological factors exert a greater influence on sexual function during pregnancy.

The prevalence of SD in the postmenopausal period varies between 65.2% and 88.7% [28,29]. In the studies, it was observed that there was a significant decrease in all subscale scores of the FSFI [28]. Smoking, advanced age, low education level, and socioeconomic inadequacies have been reported to negatively affect sexual desire, arousal, lubrication, and sexual satisfaction [30,31]. Diabetes mellitus and cardiovascular diseases have been reported to significantly impair areas of sexual function such as desire, lubrication, and satisfaction [5,32]. It has also been shown that high parity leads to significant impairments in sexual desire, orgasm, and satisfaction subscales [29]. In our study, the prevalence of SD during the postmenopausal period was 96%. There was no statistically significant difference between women with and without SD in terms of age (*p* = 0.051), educational level (*p* = 0.736), employment status (*p* = 1.0), socioeconomic status (*p* = 0.901), and smoking (*p* = 0.069). However, the parity count was significantly higher in the SD group (*p* = 0.009). In addition, a significant decrease in all subdomain scores of the FSFI was observed as parity and age increased (*p* < 0.001).

Research in the postmenopausal period has shown that low FSFI scores have negative effects on DASS-21. In particular, low satisfaction and desire scores have been reported to increase depression and negatively affect overall psychological health [33]. It has been reported that low arousal and lubrication increase depression and anxiety scores [34], and orgasm and satisfaction scores are strongly associated with depression [6]. It has been emphasized that low desire and arousal scores increase stress and anxiety, and that sexual function plays an important role in psychological well-being [35]. In our study, no significant relationship was found between the FSFI scores and the DASS-21 subscales of depression (*p* = 0.111) and stress (*p* = 0.350) in postmenopausal women; however, a significant negative correlation was observed with anxiety (*p* = 0.032). The finding of a significant association only with anxiety suggests that SD may be related to anxiety about loss of control and bodily changes in the postmenopausal period, whereas depression and stress, which are shaped by more chronic and multifactorial processes, may have masked these relationships.

The fact that women with SD are significantly older and have more pregnancy and childbirth experience suggests that this condition is closely related not only to biological but also to psychological and social factors. Physiologic mechanisms such as hormonal changes due to aging, pelvic floor trauma, vaginal atrophy, lack of lubrication, and vascular changes may lead to decreased sexual function [28,29]. However, these physiological processes are further complicated by social stressors such as impaired body image, parental burden, care responsibilities, and gender roles. The higher prevalence of SD among women with low education and unemployed women points to a strong relationship between social determinants of health and sexual function. Factors such as limited health literacy, low sexual autonomy, economic dependence, and inadequate help-seeking behavior may make SD difficult to recognize and manage [30,31]. On the other hand, the fact that no significant difference was found between women with and without SD in terms of classical sociodemographic variables such as age, parity, educational level, socioeconomic status, and smoking in our study may be explained by factors such as the relative homogeneity of the sample group, women’s difficulty in expressing their sexual problems due to cultural reservations, or low educational level limiting their capacity to respond to the survey questions with awareness. In addition, the fact that relational, emotional, and contextual factors are often at the root of SD may have led to the failure to establish statistically significant relationships with such classical variables.

Although the selection of participants from a single university hospital ensures that the study is representative of the local population, caution is warranted when generalizing the findings to other sociocultural contexts. Moreover, the cross-sectional design offers valuable insights into associations between variables but does not allow for causal inferences. Nevertheless, the strengths of this study offset these methodological limitations. The inclusion of women in the premenopausal, pregnancy, and postmenopausal stages provides a comprehensive and life-stage-specific perspective on women’s health. The use of internationally validated assessment tools and an adequate sample size enhance the reliability of the findings. In this study, certain individual and relational variables (such as previous psychiatric diagnoses, history of gynecological conditions, and the quality of the marital relationship) were excluded to preserve sample homogeneity and allow for a more focused and interpretable analysis based on key biopsychosocial variables. However, given that these factors may significantly influence both sexual function and psychological well-being, future research should incorporate them within multivariate and longitudinal designs. Despite its limitations, this study stands out as one of the few to investigate the interplay between sexual function and psychological health across different reproductive stages, offering a meaningful and clinically relevant contribution to the literature.

## 5. Conclusions

This study presents important findings by comprehensively evaluating the relationship between sexual function and psychological health in premenopausal, pregnant, and postmenopausal women. The results revealed that SD varied across life stages and reached its highest prevalence in the postmenopausal period. Furthermore, this relationship was found to be influenced by factors such as health status, socioeconomic level, and living conditions. Our study provides an important basis for understanding women’s life stage-specific needs and emphasizes the need for individualized assessment and treatment approaches. These findings are instructive for clinical practice and public health policies and should be supported by larger longitudinal designs in the future.

## Figures and Tables

**Table 1:** Demographic characteristics, FSFI, and DASS-21 results of the total cohort.

	Total Cohort (n = 300)			
Variables	n, %	Median (Min–Max)	Mean ± Standard Deviation	IQR
**Age (years)**		36 (18–70)	39.7 ± 14.1	36 [27.25–51]
**Gravida**		3 (0–18)	3.53 ± 2.6	3 [2–5]
**Parity**		2 (0–17)	2.62 ± 2.4	2 [1–4]
**Miscarriage**		0 (0–4)	0.35 ± 0.7	0 [0]
**Employment Status**	38 (12.6%)			
**Education**				
Primary school	111 (37%)			
High school	151 (50.3%)			
College/University	38 (12.7%)			
**Yearly income level**				
Low	6 (2%)			
Medium	154 (51.3%)			
High	140 (46.7%)			
**Smoking**	35 (11.7%)			
**Sexual Dysfunction**	240 (80%)			
**FSFI Scale**		21.6 (2–36)	18.46 ± 9.74	21.6 [8.8–25.4]
Sexual Desire		3 (1.2–6)	2.9 ± 1.3	3 [1.2–3.6]
Sexual Stimulation		3.3 (0–6)	2.7 ± 1.7	3.3 [1.2–3.9]
Lubrication		3.6 (0–6)	3.1 ± 1.9	3.6 [1.2–4.8]
Orgasm		3.6 (0–6)	3.0 ± 2.0	3.6 [0–4.4]
Satisfaction		3.8 (0.8–6)	3.6 ± 1.7	3.8 [2.4–4.8]
Pain		3.6 (0–6)	3.06 ± 2.06	3.6 [1.3–4.8]
**DASS-21 Scale**		9 (0–48)	11.19 ± 9.89	9 [3–17]
**Anxiety**		3 (0–16)	3.57 ± 3.4	3 [1–5]
Normal	170 (56.7%)			
Mild	57 (19%)			
Medium	32 (10.7%)			
Severe	21 (7%)			
Very Severe	20 (6.7%)			
**Depression**		2 (0–18)	3.21 ± 3.4	2 [0–5]
Normal	218 (72.7%)			
Mild	38 (12.7%)			
Medium	32 (10.7%)			
Severe	7 (2.3%)			
Very Severe	5 (1.7%)			
**Stress**		3 (0–18)	4.41 ± 4.08	3 [1–7]
Normal	235 (78.3%)			
Mild	23 (7.7%)			
Medium	29 (9.7%)			
Severe	11 (3.7%)			
Very Severe	2 (0.7%)			

**DASS-21:** Depression, Anxiety and Stress Scale-21, **FSFI:** Female Sexual Function Index, **IQR:** Interquartile Range.

**Table 2:** Comparison of those with and without sexual dysfunction.

Variables	Those Without SD (n = 60)	Those with SD (n = 240)	*p* Value
**Age (years) (Median (Min–Max))**	28 (18–53)	40.5 (18–70)	<0.001
**Gravida (Median (Min–Max))**	2 (0–7)	4 (0–18)	<0.001
**Parity (Median (Min–Max))**	1 (0–6)	3 (0–17)	<0.001
**Miscarriage (Median (Min–Max))**	0 (0–3)	0 (0–4)	0.187
**Employment Status (n, %)**	13 (21.7%)	21 (8.8%)	0.009
**Education (n, %)**			
Primary School	17 (28.3%)	94 (39.2%)	0.024
High school	29 (48.3%)	122 (50.8%)	0.024
College/University	14 (23.3%)	24 (10%)	0.024
**Yearly income level (n, %)**			
Low	0 (0%)	6 (2.5%)	0.603
Medium	37 (61.7%)	117 (48.8%)	0.073
High	23 (38.3%)	117 (48.8%)	0.148
**Smoking (n, %)**	7 (11.7%)	28 (11.7%)	1.0
**Period (n, %)**			
Premenopausal	23 (38.3%)	77 (32.1%)	<0.001
Pregnancy	33 (55%)	67 (27.9%)	<0.001
Postmenopausal	4 (6.7%)	96 (40%)	<0.001
**DASS-21 Scale (Median (Min–Max))**	9 (0–40)	9.5 (0–48)	0.194
**Anxiety (Median (Min–Max))**	3 (0–12)	3 (0–16)	0.170
Normal	37 (61.7%)	133 (55.4%)	0.382
Mild	14 (23.3%)	43 (17.9%)	0.440
Medium	2 (3.3%)	30 (12.5%)	0.068
Severe	2 (3.3%)	19 (7.9%)	0.269
Very Severe	5 (8.3%)	15 (6.3%)	0.565
**Depression (Median (Min–Max))**	1 (0–18)	3 (0–18)	0.013
Normal	48 (80%)	170 (70.8%)	0.207
Mild	5 (8.3%)	33 (13.8%)	0.362
Medium	6 (10%)	26 (10.8%)	1.0
Severe	0 (0%)	7 (2.9%)	0.352
Very Severe	2 (3.3%)	4 (1.7%)	0.345
**Stress (Median (Min–Max))**	3 (0–14)	4 (0–18)	0.542
Normal	48 (80%)	187 (77.9%)	0.861
Mild	5 (8.3%)	18 (7.5%)	0.789
Medium	6 (10%)	23 (9.6%)	1.0
Severe	1 (1.7%)	10 (4.2%)	0.700
Very Severe	0 (0%)	2 (0.8%)	1.0

**DASS-21:** Depression, Anxiety, and Stress Scale-21, **SD:** Sexual Dysfunction.

**Table 3:** Comparison of demographic and obstetric characteristics according to premenopausal, pregnancy, and postmenopausal periods.

Variables	Premenopausal (n = 100)	Pregnancy (n = 100)	Postmenopausal (n = 100)	*p* Value
**Age (years) (Median (Min–Max))**	33 (18–54)	27 (18–41)	55 (44–70)	<0.001
**Gravida (Median (Min–Max))**	3 (0–10)	2 (1–6)	5 (0–18)	<0.001
**Parity (Median (Min–Max))**	2 (0–6)	1 (0–4)	4 (0–17)	<0.001
**Miscarriage (Median (Min–Max))**	0 (0–4)	0 (0–3)	0 (0–4)	0.301
**Employment Status (n, %)**	14 (14%)	16 (16%)	4 (4%)	0.008
**Education (n, %)**				
Primary school	72 (72%)	0 (0%)	39 (39%)	<0.001
High school	13 (13%)	81 (81%)	57 (57%)	<0.001
College/University	15 (15%)	19 (19%)	4 (4%)	<0.001
**Yearly income level (n, %)**				
Low	3 (3%)	0 (0%)	3 (3%)	0.253
Medium	63 (63%)	49 (49%)	42 (42%)	0.010
High	34 (34%)	51 (51%)	55 (55%)	0.007
**Smoking (n, %)**	17 (17%)	6 (6%)	12 (12%)	0.045

**Table 4:** Comparison of FSFI scale results according to premenopausal, pregnancy, and postmenopausal periods.

Variables	Premenopausal (n = 100)	Pregnancy (n = 100)	Postmenopausal (n = 100)	*p* Value
**FSFI Scale (Median (Min–Max))**	22.1 (2–35.4)	24.25 (2–36)	4.4 (2–31.7)	<0.001
Sexual Desire	3.6 (1.2–6)	3.6 (1.2–6)	1.2 (1.2–6)	<0.001
Sexual Stimulation	3.6 (0–5.7)	3.6 (0–6)	0.6 (0–5.4)	<0.001
Lubrication	3.9 (0–6)	4.2 (0–6)	0 (0–5.4)	<0.001
Orgasm	4 (0–6)	4 (0–6)	0 (0–6)	<0.001
Satisfaction	4.4 (0.8–6)	4.8 (0.8–6)	2.4 (0.8–6)	<0.001
Pain	3.6 (0–6)	4 (0–6)	0 (0–6)	<0.001

**FSFI:** Female Sexual Function Index.

**Table 5:** Comparison of DASS-21 scale results according to premenopausal, pregnancy, and postmenopausal periods.

Variables	Premenopausal (n = 100)	Pregnancy (n = 100)	Postmenopausal (n = 100)	*p* Value
**DASS-21 Scale (Median (Min–Max))**	10 (0–48)	9 (0–45)	9 (0–44)	0.227
**Anxiety (Median (Min–Max))**	3 (0–15)	3 (0–18)	3 (0–16)	0.773
Normal	58 (58%)	56 (56%)	56 (56%)	0.947
Mild	18 (18%)	23 (23%)	16 (16%)	0.435
Medium	9 (9%)	9 (9%)	14 (14%)	0.431
Severe	8 (8%)	6 (6%)	8 (8%)	0.857
Very Severe	7 (7%)	6 (6%)	6 (6%)	0.812
**Depression (Median (Min–Max))**	3 (0–18)	2 (0–14)	2,5 (0–18)	0.144
Normal	74 (74%)	71 (71%)	73 (73%)	0.889
Mild	10 (10%)	14 (14%)	14 (14%)	0.607
Medium	11 (11%)	10 (10%)	11 (11%)	0.965
Severe	3 (3%)	4 (4%)	0 (0%)	0.168
Very Severe	2 (2%)	2 (2%)	2 (2%)	1.0
**Stress (Median (Min–Max))**	4 (0–16)	2.5 (0–18)	3 (0–15)	0.080
Normal	77 (77%)	75 (75%)	83 (83%)	0.351
Mild	7 (7%)	9 (9%)	7 (7%)	0.832
Medium	9 (9%)	14 (14%)	6 (6%)	0.155
Severe	7 (7%)	0 (0%)	4 (4%)	0.018
Very Severe	0 (0%)	2 (2%)	0 (0%)	0.331

**DASS-21:** Depression, Anxiety and Stress Scale-21.

**Table 6:** Univariate Analysis for Risk Factors for SD.

Variables	OR	95% CI	*p* Value
**Age**	1.075	1.045–1.106	<0.001
**Parity**	1.689	1.382–2.065	<0.001
**Smoking**	1.0	0.414–2.414	1.0
**Employment Status**	0.347	0.162–0.741	0.006
**Education**	0.579	0.377–0.888	0.012
**Yearly income level**	1.316	0.777–2.229	0.307
**Period**			
Premenopausal	0.760	0.423–1.366	0.359
Pregnancy	0.317	0.177–0.567	<0.001
Postmenopausal	9.333	3.277–26.584	<0.001

**CI:** confidence interval, **SD:** Sexual Dysfunction, **OR:** odds ratio.

**Table 7:** Multivariate Analysis for Risk Factors for SD.

Variables	OR	95% CI	*p* Value
**Age**	1.019	0.967–1.074	0.473
**Parity**	1.421	1.085–1.861	0.011
**Employment Status**	0.358	0.082–1.563	0.172
**Education**	1.497	0.590–3.799	0.396
**Pregnancy**	0.744	0.291–1.903	0.537
**Postmenopausal**	0.464	0.097–2.226	0.337

**CI:** confidence interval, **SD:** Sexual Dysfunction, **OR:** odds ratio.

## Data Availability

All data related to the study were included in the article.
